# Serum 25(OH) Vitamin D levels is not associated with disability in multiple sclerosis patients: A case-control study

**Published:** 2015-01-05

**Authors:** Masoud Nikanfar, Ali Akbar Taheri-Aghdam, Maria Yazdani, Sheida Shaafi, Nooshin Masoudian, Hossein Akbari, Parisa Youhanaee, Hamzeh Abbaszadeh

**Affiliations:** 1 Department of Neurology, Neuroscience Research Center, Imam Reza Hospital, Tabriz University of Medical Sciences, Tabriz, Iran; 2 Department of Neurology, School of Medicine, Tabriz University of Medical Sciences, Tabriz, Iran; 3 Department of Nutrition, School of Nutrition and Health, Tabriz University of Medical Sciences, Tabriz, Iran

**Keywords:** Serum 25(OH) vitamin D level, Disability, Multiple sclerosis

## Abstract

**Background: **It seems that serum vitamin D levels are one of the potential environmental factors affecting the severity of multiple sclerosis (MS). In this study, we aim to evaluate vitamin D levels in MS patients and healthy subjects and assess the relationship between vitamin D level and disability.

**Methods:** In this case-control study, 168 rapid relapsing MS patients and 168 matched healthy controls were randomly included in this study. Demographic characteristics and serum vitamin D levels for patients and controls, as well as expanded disability status scale (EDSS), duration of disease and diagnostic lag for patients were evaluated. We followed up patients for 6 months and relapses were recorded.

**Results: **The mean serum vitamin D levels were 19.16 ± 17.37 inpatients and 25.39 ± 19.67 in controls (P = 0.560). The mean serum vitamin D levels were 12.65 ± 13.3 in patients with relapses and 22.08 ± 18.22 in patients without any relapses (P < 0.001). There was no significant correlation between EDSS score and serum vitamin D levels (r = −0.08, P = 0.280). There was a significant positive correlation between EDSS score and disease duration (r = 0.52, P < 0.001).

**Conclusion: **In conclusion, vitamin D level in patients with MS was significantly lower than the healthy subjects, but no significant relationship was found between vitamin D levels and disability. Our findings did not suggest a protective role for serum vitamin D levels against disability.

## Introduction

Multiple sclerosis (MS) is one of the most common neurological diseases affecting adults.^1^ It is regarded as a chronic, inflammatory autoimmune disease of the central nervous system, with serious debilitating effects, which result in extensive and major economic and social impressions.^2^^-^^6^ MS has either a progressive or relapsing-remitting (RRMS) nature and manifests as acute focal inflammatory demyelination causing axonal damages.^2^^,^^6^ It usually involves young adults (20-40 years old) and has a twofold influence on women compared with men.^7^^,^^8^

There is a probable autoimmune etiology for MS, but it seems that genetic and environmental factors have an equal role in constructing the final clinical picture.^9^ Vitamin D deficiency, which seems to be a risk factor for some systemic diseases such as lupus erythematous,^10^ has been known to be modifiable risk factor for MS^11^ and recent studies suggests that vitamin D is an important environmental factor affecting the disease.^12^ On the other hand, the prevalence of MS is variable in different degrees of latitude, with a higher prevalence of the disease in high-latitude areas and vice versa. This variety is believed to be due to ultraviolet (UV) light exposure and subsequent change in vitamin D synthesis,^13^ as the dominant source of vitamin D for most people is through skin exposure to sunlight.^14^

There have been some inconsistent reports in regards to different 25(OH) vitamin D serum levels in MS patients and community controls^15^^-^^21^ and the relation of vitamin D levels and disease severity, relapse rate and disability.^6^^,^^17^^,^^22^^-^^24^ It has been shown in some studies that high levels of 25(OH) vitamin D is related with lower risk of relapsing and lower disability expressed as expanded disability status scale (EDSS) score.

There are some reports about increasing incidence of MS in Middle Eastern countries, including Iran.^25^^-^^28^ The incidence of MS has been increased from 3.64 person per 100000 population in 2007 to 9.1 person per 100000 population in 2009.^26^ Considering the relatively high prevalence of MS in Iran,^29^^,^^30^ higher latitude in North West of Iran than other provinces and uncertainty about the role of serum vitamin D levels in the severity of MS, we conducted this study to compare the serum level of 25-hydroxy vitamin D in MS patients with healthy controls and to investigate its potential relation with disability and relapse rate in our patients.

## Materials and Methods

This case–control study was designed and performed in the Neurology Department of Razi Hospital, Tabriz University of Medical Sciences, Tabriz, Iran. Between June 2012 November 2012, 168 definitive MS patients of East Azarbaijan MS society with RRMS were enrolled in the study. RRMS was confirmed by clinical findings and magnetic resonance imaging. Inclusion criteria were disease duration based on the initiation of symptoms for at least 6 months, being in the remission phase without any history of a new attack in the last month, no history of diseases related to vitamin D deficiency and no intake of drugs or supplements containing vitamin D in last 30 days.^31^ One hundred and seventy-five healthy controls from Razi and Imam Reza Hospitals staff matched for age, gender and time and date of blood sampling were included in the study. Only those patients and healthy subjects were evaluated with available follow-up data. Informed consent was obtained from all participants. Blood samples were obtained after an overnight fasting and were measured by chemiluminescent immunoassay method for 25(OH) vitamin D levels, all in the same laboratory. Demographic characteristics, including age and gender, family history of MS in first and second degree family members, duration of disease from the first symptoms presentation, diagnostic lag, EDSS score and relapse rate were recorded by one neurologist. After taking blood samples, patients were followed up monthly for 6 months by phone calls and relapses were recorded based on both patients self-reports and medical documentation. We excluded patients whom we were unable to contact in follow-up period. Corresponding controls were excluded too. We defined Vitamin D deficiency, insufficiency, and normal status as 25(OH)D levels < 10 ng/ml, between 10 and 30 ng/ml and more than 30 ng/ml, respectively.

Statistical analyses were performed using the SPSS for Windows (version 17.0, SPSS, Chicago, Illinois, USA). Quantitative data were presented as mean ± standard deviation, whereas qualitative data were demonstrated as frequency and percent (%). Demographic data, clinical parameters and laboratory values of the patients were compared with controls, using the chi-square and Student’s t-test methods, as appropriate. Pearson’s correlation analysis was used to determine the relationship between serum vitamin D levels and duration of disease, EDSS score, diagnostic lag and age. A P < 0.050 was considered as significant.

## Results

In this study, a total of 168 MS patients and 168 matched controls for age and sex were studied. The demographic and clinical characteristics of both groups are summarized in table 1. RRMS patients had significantly lower serum vitamin D levels, higher vitamin D deficiency and less Regular full-time jobs compared to healthy controls. In RRMS patients, mean duration of disease (from first symptoms initiation) and diagnostic lag were 7.41 ± 4.80 and 1.32 ± 1.92 years respectively in patients. Mean EDSS was 2.83 ± 1.18 and mean relapse rate in the 6 months follow-up period was 1.28 ± 0.45 in patients. Fifty-two patients (31%) had at least one episode of relapse in a 6 months period after taking blood samples.

**Table 1:** Demographic and clinical characteristics of patients and controls

**Characteristics**	**Patients (n = 168) (%)**	**Controls (n = 168) (%)**	**P **
Female (%)	131 (78)	127 (75.6)	0.880
Age^*^	33.6 ± 7.69	34.43 ± 7.31	0.620
Serum Vitamin D^*^	19.16 ± 17.37	25.39 ± 19.67	0.002^*^
Vitamin D status			
Deficiency	80 (47.62)	49 (29.17)	0.002^*^
Insufficiency	53 (31.55)	68 (40.48)	
Normal	35 (20.83)	51 (30.35)	

P is two-sided significant;

* Numbers are provided as mean ± standard deviation

**Table 2:** Demographic and clinical characteristics of patients with and without relapses in follow up period

**Variable**	**Relapses (52)**	**No relapses (116)**	**P **
Female (%)	41 (78.8)	90 (77.6)	0.850
Family history of MS	13 (25)	26 (22.4)	0.710
Serum Vitamin D*	12.65 ± 13.30	22.08 ± 18.22	< 0.001*
Age*	34.09 ± 6.92	33.37 ± 8.03	0.570
EDSS*	3.00 ± 0.76	2.75±1.33	0.240
Disease duration*^,^**	7.56 ± 4.65	7.42 ± 5.16	0.670
Diagnostic lag*^, ^***	1.63 ± 2.29	1.18 ± 1.72	0.160

P is two-sided significant;

* Numbers are provided as mean ± standard deviation;

** Duration of disease from the first symptoms presentation;

*** Delay between symptoms presentation and definite diagnosis; EDSS: Expanded disability status scale

**Table 3:** Correlations between independent variables and expanded disability status scale (EDSS)

**Variable**	**Coefficient**	**P**
Sex	-0.05	0.440
Age	0.29	< 0.001*
Serum vitamin D	-0.08	0.280
Disease duration	0.52	< 0.001*
Diagnostic lag	0.19	0.010*

* P < 0.050 is considered significant

RRMS patients were divided into with and without relapse (Table 2). There was no difference between two groups regarding the age, gender, family history of MS, EDSS score, disease duration and diagnostic lag. However, serum vitamin D levels were significantly lower in patients with relapse compared with no relapse patients. Although serum vitamin D levels were insignificantly lower in female patients (20.09 ± 18.23 in males and 18.9 ± 17.19 in females), but there was no difference between male and female patients regarding to parameter studied such as, EDSS score and diagnostic lag.

Table 3 demonstrates the correlation between quantitative values and EDSS. There was significantly positive correlation between age, disease duration and diagnostic lag with EDSS. We observed no significant correlation between EDSS score and serum vitamin D levels and patients’ gender.

We also compared vitamin D levels between genders in two groups. Serum 25(OH)D was slightly lower in female patients and controls compared to males, but this difference was not statistically significant (20.09 ± 18.23 in males and 18.9 ± 17.19 in females (P = 0.710) in cases and 29/87 ± 20.48 in males and 25.2 ± 19.12 in females (P = 0.210) in control group). 

## Discussion

MS risk associated with low vitamin D levels might vary between ethnicities and regions with different latitude; For example, it was reported that the MS risk significantly decreased with increasing 25(OH)D serum levels in Caucasians, whereas no significant associations between 25(OH)D levels and MS risk were found among Africans and Hispanics.^11^ Furthermore, there is some inconsistency between different reports; Correale et al.^19^ evaluated serum 25(OH)D levels between Spanish MS patients and healthy controls and reported a significant lower serum 25(OH)D levels compared to control group. In contrast to this study, Kragt et al.^20^ followed up MS patients and healthy subjects for a year by performing a large cohort in The Netherlands and reported no difference between two groups throughout the whole follow up period.

To the best of our knowledge, no study has been carried out in Northwestern of Iran. In this study, we investigated levels of serum vitamin D in MS patients and compared it with matched healthy controls. We also studied possible correlation between the severity of the disease (EDSS score) and other evaluated parameters. Findings of this study suggest that serum Vitamin D levels are significantly lower in MS patients compared to healthy subjects, but there is no correlation between this laboratory finding and disease’s severity. We found that there is a significant positive correlation between EDSS score and patients’ age and disease duration.

Our results had some similarities and differences to recent findings. Similar to most of previous studies^19^^,^^21^^,^^22^^,^^31^^,^^32^ and unlike Kragt et al.^20^ and van der Mei et al.^17^ we found that serum vitamin D levels are significantly different between patients and matched control. Regarding to substantial strength given to the hypothesis and higher latitude and lower temperature of North Western of Iran compared to equatorial regions^17^ and central provinces of Iran^21^ and overlooking the different criteria used in various studies to define vitamin D deficiency and insufficiency, it seems that vitamin D deficiency is an important phenomenon in MS patients which may occur due to different reasons such as decreased outdoor activity and exposure to UVB sunlight, as the role of sunlight exposure in vitamin D synthesis is definitive.^33^

In this study, serum 25(OH)D was slightly lower in female patients and controls compared to males that can be due to heavier cloth cover used by female ones in Iranian society. Moreover, it must be noted that some of these differences may be as a result of non-fasting sampling and different methods for measuring serum vitamin D,^17^ low recruited participants^16^^,^^18^ and unmatched groups.^20^

Our results showed that there is no significant correlation between EDSS and serum vitamin D levels. Most of the recent efforts show that EDSS is directly correlated with serum vitamin D levels. van der Mei et al.^17^ studied 127 MS patients and showed that patients with EDSS more than 3 are more likely to have lower vitamin serum concentrations. Smolders et al.^23^ reported a significant positive correlation between EDSS and serum vitamin D levels. Harandi et al.^34^ studied 78 Iranian MS patients and reported such relationship between theses to parameters only in female patients. Unlike these studies, Yildiz et al.^35^ and Hatamian et al. didn’t present any significant relation between EDSS and serum vitamin D status. They presumed small study population as a probable reason for an insignificant relation. Considering large study population in this survey, different skin types, exact geographical location, socioeconomic status, lower disease duration and EDSS score compared to other studies and genetic variation are potential factors affecting this correlation. Although it is reported that replacement supplement therapy has equal effect as placebo therapy on EDSS score and relapse rate in MS patients, but it looks that vitamin D replacement in these patients should be considered in their therapeutic and follow up plan and despite their disability, it can be beneficial for their resistance to mechanical traumas and reducing fractures that could affect patient’s outdoor activity and sunlight exposure.

## Conclusion

Vitamin D level in patients with MS was significantly lower than the healthy subjects, but no significant relationship was found between vitamin D level and disability.

## Notes

**How to cite this article: **Nikanfar M, Taheri-Aghdam AA, Yazdani M, Shaafi Sh, Masoudian N, Akbari H, et al. Serum 25(OH) Vitamin D levels is not associated with disability in multiple sclerosis patients: A case-control study. Iran J Neurol 2015; 14(1): 17-21.
